# Supplementary figures and images for: Development and validation of a novel survival model for acute myeloid leukemia based on autophagy-related genes
Source: PeerJ. 2021 Aug 12;9:e11968. doi: 10.7717/peerj.11968 (PMC8364747; doi:10.7717/peerj.11968)

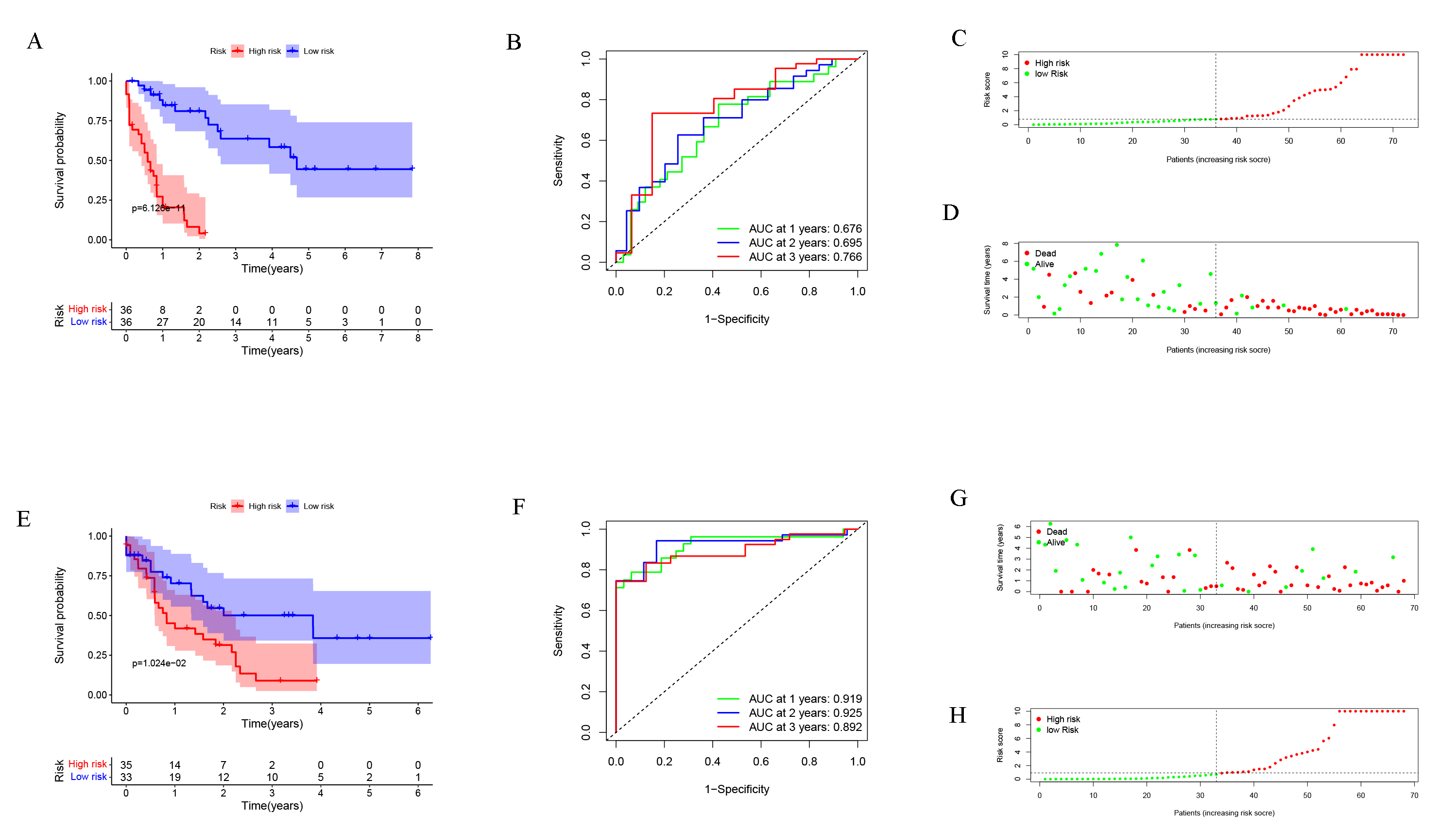

Supplement: Supplemental Information 1 — The training set randomly divide into a verification 2 set (Fig. S1A–D) and a verification 3 set (Fig. S1E-H). [file peerj-09-11968-s001.png]

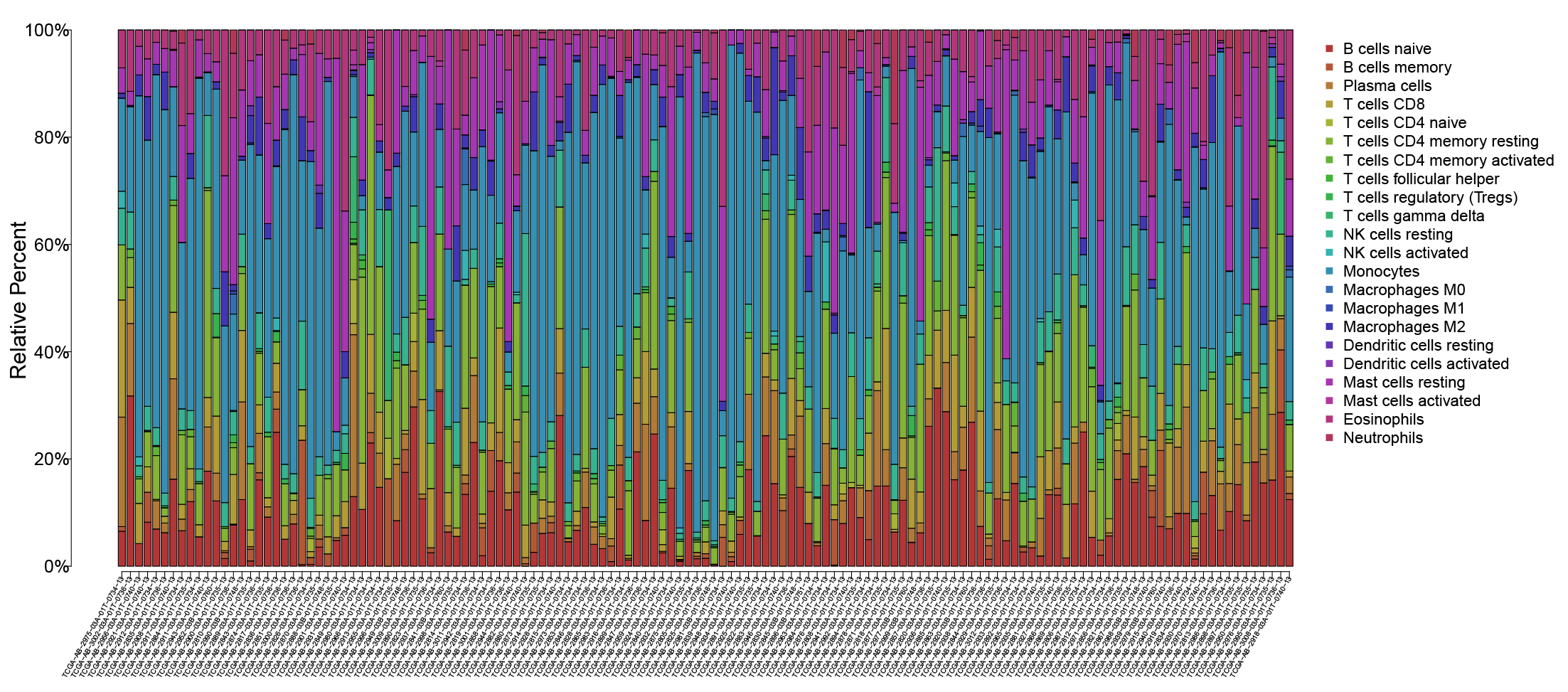

Supplement: Supplemental Information 2 [file peerj-09-11968-s002.png]

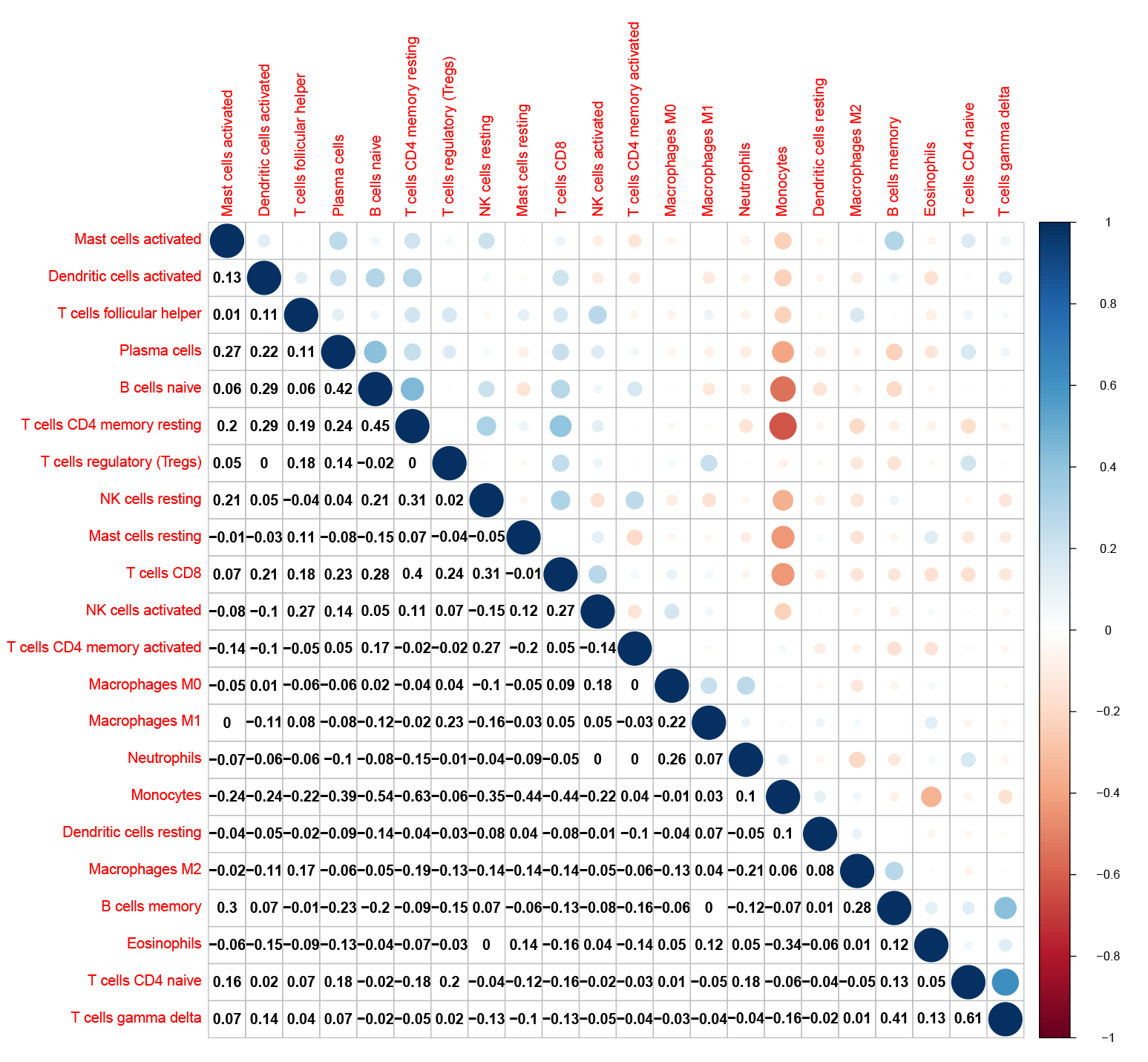

Supplement: Supplemental Information 3 [file peerj-09-11968-s003.png]
