# Supplementary material for: Development and validation of a novel survival model for acute myeloid leukemia based on autophagy-related genes
Source: PeerJ. 2021 Aug 12;9:e11968. doi: 10.7717/peerj.11968 (PMC8364747; doi:10.7717/peerj.11968)
Supplement: Supplemental Information 4 [file peerj-09-11968-s004.docx]

**TABLE S1 Autophagy-related genes**

| Autophagy-related genes |  |
| --- | --- |
|  | AMBRA1, APOL1, ARNT, ARSA, ARSB, ATF4, ATF6, ATG10, ATG12, ATG16L1, ATG16L2, ATG2A, ATG2B, ATG3, ATG4A, ATG4B, ATG4C, ATG4D, ATG5, ATG7, ATG9A, ATG9B, ATIC, BAG1, BAG3, BAK1, BAX, BCL2, BCL2L1, BECN1, BID, BIRC5, IRC6, BNIP1, BNIP3, BNIP3L, C12orf44, C17orf88, CALCOCO2, CAMKK2, CANX, CAPN1, CAPN10, CAPN2, CAPNS1, CASP1, CASP3, CASP4, CASP8, CCL2, CCR2, CD46, CDKN1A, CDKN1B, CDKN2A, CFLAR, CHMP2B, CHMP4B, CLN3, CTSB, CTSD, CTSL1, CX3CL1, CXCR4, DAPK1, DAPK2, DDIT3, DIRAS3, DLC1, DNAJB1, DNAJB9, DRAM1, EDEM1, EEF2, EEF2K, EGFR, EIF2AK2, EIF2AK3, EIF2S1, EIF4EBP1, EIF4G1, ERBB2, ERN1, ERO1L,FADD, FAM48A, FAS, FKBP1A, FKBP1B, FOS, FOXO1, FOXO3, GAA, GAA, GABARAP, GABARAP, GABARAPL1, GABARAPL1, GABARAPL2, GABARAPL2, GAPDH, GAPDH, GNAI3, GNAI3, GNB2L1, GNB2L1, GOPC, GOPC, GRID1, GRID1, GRID2, GRID2, HDAC1, HDAC6, HGS, HIF1A, HSP90AB1, HSPA5, HSPA8, HSPB8, IFNG, IKBKB, IKBKE, IL24, IRGM, ITGA3, ITGA6, ITGB1, ITGB4, ITPR1, KIAA0226, KIAA0652, KIAA0831, KIF5B, KLHL24, LAMP1, LAMP2, MAP1LC3A, MAP1LC3B, MAP1LC3C, MAP2K7, MAPK1, MAPK3, MAPK8, MAPK8IP1, MAPK9, MBTPS2, MLST8, MTMR14, MTOR, MYC, NAF1, NAMPT, NBR1, NCKAP1, NFE2L2, NFKB1, NKX2-3, NLRC4, NPC1, NRG1, NRG2, NRG3, P4HB, PARK2, PARP1, PEA15, PELP1, PEX14, PEX3, PIK3C3, PIK3R4, PINK1, PPP1R15A, PRKAB1, PRKAR1A, PRKCD, PRKCQ, PTEN, PTK6, RAB11A, RAB1A, RAB24, RAB33B, RAB5A, RAB7A, RAC1, RAF1, RB1, RB1CC1, RELA, RGS19, RHEB, RPS6KB1, RPTOR, SAR1A, SERPINA1, SESN2, SH3GLB1, SIRT1, SIRT2, SPHK1, SPNS1, SQSTM1, ST13, STK11, TBK1, TM9SF1, TMEM49, TMEM74, TNFSF10, TP53, TP53INP2, TP63, TP73, TSC1, TSC2, TUSC1, ULK1, ULK2, ULK3, USP10, UVRAG, VAMP3, VAMP7, VEGFA,WDFY3, WDR45, WDR45L, WIPI1, WIPI2, ZFYVE1 |
