# Supplementary material for: Development and validation of a novel survival model for acute myeloid leukemia based on autophagy-related genes
Source: PeerJ. 2021 Aug 12;9:e11968. doi: 10.7717/peerj.11968 (PMC8364747; doi:10.7717/peerj.11968)
Supplement: Supplemental Information 5 [file peerj-09-11968-s005.docx]

**TABLE S2 The clinical information of the patients.**

| Characteristic | Training set | verification 1 set | verification 2 set | verification 3 set |
| --- | --- | --- | --- | --- |
| Survival status |  |  |  |  |
| Alive | 57 (40.7%) | 147 (26.6%) | 33 (45.8%) | 24 (35.3%) |
| Dead | 83 (59.3%) | 406 (73.4%) | 39 (54.2%) | 44 (64.7%) |
| age |  |  |  |  |
| >65 | 29 (20.7%) | 160 (28.9%) | 19 (26.4%) | 10 (14.7%) |
| <=65 | 111 (79.3%) | 393 (71.1%) | 53 (73.6%) | 58 (85.3%) |
| gender |  |  |  |  |
| MALE | 76 (54.3%) | NA | 39 (54.2%) | 37 (54.4%) |
| FEMALE | 64 (45.7%) | NA | 33 (45.8%) | 31 (45.6%) |
